# Bifurcation structure of phase locked modes in Type I excitable cells based on phase and spike time resetting curves

**DOI:** 10.1186/1471-2202-13-S1-P78

**Published:** 2012-07-16

**Authors:** Sorinel A Oprisan

**Affiliations:** 1 Department of Physics and Astronomy, College of Charleston, Charleston, SC 29424, USA

## 

**Figure 1:**
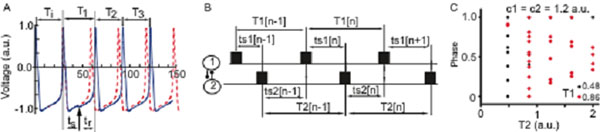
(A) A presynaptic perturbation (vertical arrow) delivered at stimulus time t_s_ changes the intrinsic firing period T_i_ to T_1_, T_2_, T_3_, … The unperturbed activity (dashed line) is shifted (reset) due to an external perturbation. (B) In a two-neuron network, when neuron “2” fires (solid rectangle), it perturbs neuron “1” at a stimulus time t_s1_[n-1], during (n-1)^th^ cycle. In turn, neuron “1” recovers from that perturbation and fires again after T_1_[n-1] perturbing neuron “2” at stimulus time t_s2_[n]. (C) In its canonical form, the PRC of a Type I excitable cell only depends on the strength of coupling between cells and the intrinsic firing periods, which leads to a four-dimensional parameter space for a two-neuron network: (c_1_, c_2_, T_1_, T_2_). The recursion (1) predicts the steady phase locked mode.

Some neurons are endogenous bursters, i.e., they intrinsically fire with a steady period (*T_i_*) in the absence of external stimuli. In neural networks, the neurons constantly receive stimuli, *e.g.*, presynaptic inputs that perturb their intrinsic activity. The transient changes in the firing frequency of a neuron due to a presynaptic input received at a stimulus time (*t_s_*), or phase *φ* = *t_s_/T_i_*, is tabulated by the phase resetting curve (PRC). The first order PRC is extracted in open loop, *i.e.*, from isolated neurons, by tabulating the relative change in the firing period *F*(*φ*) = *∆T_1_/T_i_* = (*T_1_-T_i_*)*/T_i_* due to a stimulus received during the current cycle at phase φ (see Fig. 1A). Equivalently, we use the spike time resetting curve (STRC) that tabulates the recovery time *t_r_* of a neuron that received an input during the current cycle at a stimulus time *t_s_* (see Fig. 1A). The assumption is that the open loop PRC/STRC can predict the phase locked modes of a network provided, among other requirements, that the open loop stimulus closely resembles the input the neuron receives in a fully connected network. For example, the PRC/STRC method can predict the 1:1 phase locked modes of a two-neuron network (see Fig. 1B). Assuming that the effect of the presynaptic input dies out by the time the neuron receives another input, we can derive (Figure 1B) the recursions that determine the phase locked mode:(1)

which can be translated into phase recursions by using the definitions φ = t_s_/T_i_ and T_1_(φ) = T_i_(1+F(φ)). Very close to the bifurcation point of a Type I excitable cell, the infinitesimal PRC is F(φ) = cT_i_(1-cos(φ)), where c is a model-dependent constant [1,2]. We explored the bifurcation structure of phase locked modes of two coupled Type I intrinsic bursters (see a particular example of bifurcation diagram in Figure 1C). We also derived stability criteria for predicted phase locked modes. The advantage of the proposed approach is that it reveals the route to phase locked mode transition. The challenge of the method is representing efficiently a high dimensional solution in two dimensions. For this purpose, we used the method of multidimensional stacks.
